# Association of shared decision-making with financial toxicity and coping actions in adult patients with cancer: a nationwide study in China

**DOI:** 10.1093/oncolo/oyae065

**Published:** 2024-09-01

**Authors:** Meicen Liu, Dengmin Huang, Jing Ma, Wenqiang Wei, Yuanli Liu

**Affiliations:** National Cancer Center/National Clinical Research Center for Cancer/Cancer Hospital, Chinese Academy of Medical Sciences and Peking Union Medical College, Beijing 100021, People’s Republic of China; School of Health Policy and Management, Chinese Academy of Medical Sciences and Peking Union Medical College, Beijing 100730, People’s Republic of China; Institute of Hospital Management, Tsinghua University, Shenzhen 518055, People’s Republic of China; National Cancer Center/National Clinical Research Center for Cancer/Cancer Hospital, Chinese Academy of Medical Sciences and Peking Union Medical College, Beijing 100021, People’s Republic of China; School of Health Policy and Management, Chinese Academy of Medical Sciences and Peking Union Medical College, Beijing 100730, People’s Republic of China

**Keywords:** financial toxicity, shared decision-making, decision support, cancer patient, China

## Abstract

**Objective:**

Evidence on the impact of shared decision-making (SDM) on the financial toxicity (FT) of patients with cancer in real clinical settings is lacking. Using a nationwide patient survey in China, we aimed to identify the prevalence of SDM, FT as well as its consequent coping actions, and investigate their associations in adult patients with cancer.

**Methods:**

A cross-sectional survey was administered to patients with cancer near discharge at 33 tertiary public cancer hospitals across China between January and March 2021. The FT was measured using the COST (comprehensive score for financial toxicity) tool and was categorized into 3 groups: no (COST 26-44), mild (COST 14-25), and moderate or severe (COST 0-13) FT. SDM was measured using 2 items from patient’s perspective. The surveyed questionnaire also included patient’s coping actions, demographics, and clinical information. Multinomial logistic regression was used to investigate the association between SDM and FT, and for sensitivity analysis, linear regression was also used taking the COST score as a dependent variable. Pearson’s chi-square tests were used for association analyses of coping actions with FT and SDM.

**Results:**

A total of 5008 adult patients with cancer were included, of which 26.18%, 54.13%, and 19.69% patients were categorized as no FT, mild FT, and moderate or severe FT with the average COST score of 21.0 for all patients. Besides, 3943 (78.73%) patients reported a better SDM. Better SDM had significantly lower odds of developing high FT than worse SDM (adjusted OR_mild vs no_ 0.50, 95% CI 0.39-0.56; adjusted OR_moderate or severe vs no_ 0.63, 95% CI 0.50-0.80). In sensitivity analysis, better SDM was significantly associated with higher COST scores (β: 1.34, 95% CI 0.85-1.83), which indicated lower FT. Patients with higher FT were more likely to take adverse coping actions including treatment nonadherence, limiting basic health service expense, reducing leisure activity expense, and loan.

**Conclusions:**

Adult patients with cancer reporting worse SDM tended to experience higher FT and those with higher FT tended to take adverse coping actions. Understanding the impact of SDM on FT is crucial to inform early interventions designed to mitigate FT and consequent coping actions in cancer care practice.

## Teaser

Using a nationwide patient survey in China, this study focused on the prevalence of shared decision-making, financial toxicity as well as the consequent coping actions, and their associations in adult patients with cancer.

**Implications for Practice**

Using a nationwide patient survey in China, this study found adult patients with cancer reporting worse shared decision-making tended to experience higher financial toxicity and consequently tended to take adverse coping actions. Understanding the impact of patient involvement in decision-making on financial burden perception of adult patients with cancer is crucial to inform early interventions designed to mitigate financial stress and coping actions in cancer care practice.

## Background

Cancer is the leading cause of death worldwide and in China. Nearly 19.3 million new cancer cases and 10.0 million cancer deaths occurred globally in 2020, of which 24% new cases and 27% deaths occurred in China.^1^ Early diagnosis and appropriate treatment are effective in increasing survival and prolonging life for patients with cancer, while heavy financial burden limited accessibility and utilization of appropriate care for patients with cancer.^2^ A rapid rise in health care costs and high out-of-pocket spending for patients with cancer is occurring due to innovative medication and therapy in China.^3^ A multicenter retrospective survey conducted in China found total expenditures for health services of patients with colorectal cancer increased twice from 2005 to 2014.^4^ Previous surveys also showed over one-third households due to cancer care experienced catastrophic health expenditure in China, especially in rural and remote areas.^3,5-7^

Financial toxicity (FT) refers to subjective financial distress and perceived financial burden involving medical care cost, which can be quantified using the COST (comprehensive score for financial toxicity) tool.^8-10^ In the last decade, studies emerged about FT’s prevalence, risk factors, and downstream consequences on patient outcomes in many middle- and high-income countries and some low-income countries.^11-15^ Studies in China described a high prevalence of FT for patients with cancer, identified patient characteristics of at-risk groups and adverse coping actions from patients with cancer and their families, such as treatment nonadherence or delays of needed care, reduced primary care services utilization and decreased quality of household living.^15-18^ Solutions and mitigation strategies need urgent focus in order to address FT in cancer care.

Shared decision-making (SDM), a kind of potential intervention strategy that oncologists and health systems can take to mitigate FT.^19^ It works by involving clinicians and patients, sharing the best available evidence and decision support, and recognizing the needs, values, and preferences of individuals and their families.^20,21^ The American Society of Clinical Oncology (ASCO) also has recommended clinicians take the costs of cancer care into treatment discussion.^22^ A recent review outlined 4 potential strategies to mitigate FT including FT screening, financial navigation and assistance, incorporating cost information into treatment decision-making, as well as minimizing low-value care, while it revealed deficient implementation of these interventions in clinical practice.^19^ Another review also found the effectiveness of intervention strategies in mitigating FT across diverse health care systems was little known.^23^ An empirical study among 95 patients with metastatic breast cancer found patient-driven and shared decision trended toward lower FT comparing physician-driven decision.^24^ However, evidence from large-scale clinical practice about the status of SDM and its impact on the FT of cancer care is lacking worldwide and in China.

This nationwide cross-sectional study aimed to describe the prevalence of SDM, FT as well as coping actions among adult patients with cancer in China, and investigate their associations.

## Materials and methods

### Study design

The China National Patient Surveys, conducted annually between 2015 and 2021, were a part of evaluation program of the National Healthcare Improvement Initiative initiated by National Health Commission of the People’s Republic of China. The details of surveys have been described in previous studies.^17,25,26^ This survey regarding cancer care was based on the fifth round of China National Patient Surveys in 2021. At least one public tertiary cancer hospital for each province, autonomous region, and municipality in China was recruited and finally, 33 tertiary public cancer hospitals were included, which covered nearly half of public cancer hospitals in China. These surveyed hospitals are all comprehensive cancer centers and represent the local prominent performance of cancer care. This study was approved by the Ethics Committee of the Institute of Medical Biology of Chinese Academy of Medical Sciences (IPB-2020-23).

### Patients

**Figure 1.**
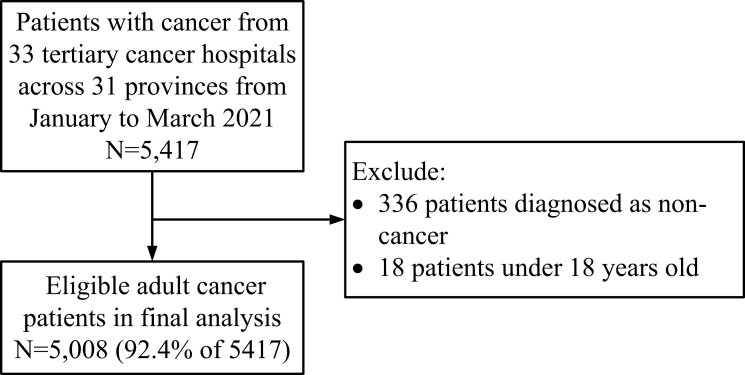
Flowchart of study population.

At least 150 inpatients to be discharged within 1-2 days and willing to participate in this study were continuously recruited from each hospital between January and March 2021. Approximately 5%-10% of inpatients in each hospital refused participation in this study. The inpatients only to be discharged were recruited because they experienced the whole process of hospitalization service and were going to pay their medical expenses, thus they have more accurate and completed perceptions of SDM and FT. During the session of investigation, a total of 5417 inpatients were interviewed by trained investigators and all of them completed the electronic questionnaire. All patients provided informed consent for their participation in the study. This study mainly focused on adult patients with cancer, thus 336 patients diagnosed as noncancer and 18 patients under 18 years old were excluded from our study. Finally, 5008 (92.4%) patients were included in our analyses (Figure 1).

### Variables and outcomes

#### Patient information

Patient characteristics included patients’ socio-demographics (gender, age, marital status, ethnicity, education, registered residency region, annual household income, household size, medical insurance, and medical assistance) and clinical information (cancer type, cancer stage, years since diagnosis, self-reported health status, and length of hospital stay).

#### Financial toxicity

Financial toxicity was quantified using the COST tool, which included 12 items rated with the 5-point Likert scale. The total score of the COST ranged from 0 to 44 points. Lower scores indicated higher FT and worse financial conditions. We categorized FT into 3 groups based on a proposed grading scale by De Souza JA: no (COST 26-44), mild (COST 14-25), and moderate or severe (COST 0-13) FT.^27^

#### Shared decision-making

In this study, SDM was assessed from patient’s perspective using 2 items: (1) clinicians carefully explained to me about treatment schemes including pros and cons and (2) clinicians took my treatment preferences into account. These 2 items were from a part of cancer patient experience scale,^26^ as an exploratory tool to measure the implemented status of SDM in clinical settings. They were designed based on the concept of SDM (clinicians should share the best available evidence and decision support, and recognize the needs, values, and preferences of patients and their families). Rounds of discussion and revisions were made by a panel of experts including oncologists, researchers, and statisticians. Each patient gave a score for every item according to their experience during this hospital stay, using a 5-point Likert scale (5—strongly agreed to 1—strongly disagreed). The correlation coefficient of 2 items was 0.63, with a sound internal consistency. SDM was classified into binary variable, defined as better SDM when the average score of 2 items was over 4, and worse SDM when the average score of 2 terms was 4 or below.

#### Coping actions

The coping actions were collected using a survey question, “have you ever taken the following actions due to financial difficulties,” followed by 7 items with “yes” or “no” responses:

1. once considered quitting treatment,
2. have delayed treatment for more than 7 days,
3. have failed to take medicine as instructed,
4. did not conduct medical checks as instructed,
5. have reduced spending on leisure activities, such as shopping or travelling,
6. have reduced spending on basic health services, such as clinic visits or vaccinations,
7. have borrowed money from relatives and friends or acquired a loan from bank due to illness.

### Statistical analysis

Descriptive statistics were used to summarize FT, SDM, coping actions, and characterize the patient population. Pearson’s chi-square tests were used to explore associations of patient characteristics with SDM and FT, as well as for associations of various coping actions with SDM and FT. Multinomial logistic regression was used to identify the association between SDM and FT with patient characteristics as covariates. Ordered logistic regression was not used because the parallel assumption was not accepted. For sensitivity analysis, we also explored the adjusted association between SDM and FT using multivariate linear regression, taking the COST score as a dependent variable. All statistical analyses were conducted with Stata/SE 15.0 software (Stata Corp LP, College Station, TX, USA). A 2-tailed *P*-value of < .05 was considered statistically significant.

## Results

### Patient characteristics

Of 5008 patients, 2358 (47.08%) patients were male, and median age for all patients was 54 (IQR: 45-63). Patients’ cancer types including lung (16.97%), esophageal (3.29%), stomach (7.21%), colorectal (10.58%), liver (3.81%), breast (13.26%), cervical (5.67%), other cancers (32.35%), etc. And 1547 (30.80%) patients had cancer stages I-II, 1685 (33.65%) patients had cancer stages III-IV, and 1776 (35.46%) patients did not know their cancer stage (Table 1).

**Table 1.** COST score and FT among Chinese patients with cancer.

Patient characteristics	*N* (%)	COST score (*x* ± *s*)	Financial toxicity				
			No	Mild	Moderate or severe	Chi-square	*P*
Total	5008 (100)	21.0 (9.30)	1311 (26.18)	2711 (54.13)	986 (19.69)	—	—
Demographics							
Gender							
Male	2358 (47.08)	20.6 ± 8.95	562 (23.83)	1328 (56.32)	468 (19.85)	13.345	.001
Female	2650 (52.92)	21.4 ± 9.58	749 (28.26)	1383 (52.19)	518 (19.55)		
Age (years)							
18-44	1177 (23.50)	21.4 ± 9.48	319 (27.10)	644 (54.72)	214 (18.18)	19.674	.003
45-54	1330 (26.56)	20.3 ± 9.53	322 (24.21)	700 (52.63)	308 (23.16)		
55-64	1422 (28.39)	20.8 ± 9.17	367 (25.81)	770 (54.15)	285 (20.04)		
65-90	1079 (21.55)	21.7 ± 8.89	303 (28.08)	597 (55.33)	179 (16.59)		
Marital status							
Married	4618 (92.21)	21.1 ± 9.27	1218 (26.38)	2511 (54.37)	889 (19.25)	7.285	.026
Single/divorced/widowed	390 (7.79)	20.0 ± 9.50	93 (23.85)	200 (51.28)	97 (24.87)		
Ethnicity							
Han	4667 (93.19)	21.1 ± 9.34	1236 (26.48)	2521 (54.02)	910 (19.50)	3.872	.144
Minority	341 (6.81)	20.1 ± 8.69	75 (21.99)	190 (55.72)	76 (22.29)		
Education							
College or more	1263 (25.22)	24.0 ± 9.30	485 (38.40)	631 (49.96)	147 (11.64)	174.116	<.001
High school	1312 (26.20)	20.9 ± 8.91	327 (24.92)	730 (55.64)	255 (19.44)		
Junior school	1457 (29.09)	19.8 ± 9.40	311 (21.35)	801 (54.98)	345 (23.68)		
Primary school or less	976 (19.49)	19.1 ± 8.70	188 (19.26)	549 (56.25)	239 (24.49)		
Registered residency region							
Urban	2818 (56.27)	22.5 ± 9.07	894 (31.72)	1508 (53.51)	416 (14.76)	155.617	<.001
Rural	2190 (43.73)	19.1 ± 9.23	417 (19.04)	1203 (54.93)	570 (26.03)		
Annual household income (1000 yuan)						500.709	<.001
<30	1395 (27.86)	17.5 ± 8.82	190 (13.62)	769 (55.13)	436 (31.25)		
30-60	1666 (33.27)	19.6 ± 8.80	325 (19.51)	964 (57.86)	377 (22.63)		
≥60	1947 (38.88)	24.7 ± 8.74	796 (40.88)	978 (50.23)	173 (8.89)		
Household size							
1-2	1217 (24.30)	21.8 ± 9.45	354 (29.09)	653 (53.66)	210 (27.26)	11.891	.018
3-4	2279 (45.51)	20.9 ± 9.33	590 (25.89)	1235 (54.19)	454 (19.92)		
≥5	1512 (30.19)	20.5 ± 9.09	367 (24.27)	823 (54.43)	322 (21.30)		
Medical insurance^1^							
UEBMI	1840 (36.74)	22.5 ± 9.16	590 (32.07)	984 (53.48)	266 (14.46)	116.211	<.001
URBMI	2860 (57.11)	19.8 ± 9.17	608 (21.26)	1580 (55.24)	672 (23.50)		
Others	308 (6.15)	22.9 ± 9.59	113 (36.69)	147 (47.73)	48 (15.58)		
Medical assistance^2^							
Yes	527 (10.52)	18.4 ± 8.74	98 (18.60)	286 (54.27)	143 (27.13)	29.524	<.001
No	4481 (89.48)	21.3 ± 9.31	1213 (27.07)	2425 (54.12)	843 (18.81)		
Clinical information							
Cancer type							
Lung	850 (16.97)	20.3 ± 9.52	196 (23.06)	462 (54.35)	192 (22.59)	49.738	<.001
Esophageal	165 (3.29)	20.7 ± 7.96	46 (27.88)	92 (55.76)	27 (16.36)		
Stomach	361 (7.21)	21.6 ± 8.65	95 (26.32)	214 (59.28)	52 (14.40)		
Colorectal	530 (10.58)	20.9 ± 8.72	132 (24.91)	299 (56.42)	99 (18.68)		
Liver	191 (3.81)	18.7 ± 8.38	39 (20.42)	99 (51.83)	53 (27.75)		
Breast	664 (13.26)	20.9 ± 9.33	175 (26.36)	351 (52.86)	138 (20.78)		
Cervical	284 (5.67)	21.2 ± 9.52	76 (26.76)	157 (55.28)	51 (17.96)		
Other	1620 (32.35)	21.3 ± 9.56	448 (27.65)	848 (52.35)	324 (20.00)		
Unidentified	229 (4.57)	21.1 ± 8.45	55 (24.02)	135 (58.95)	39 (17.03)		
Benign tumor	114 (2.28)	25.9 ± 10.5	49 (42.98)	54 (47.37)	11 (9.65)		
Cancer stage							
1-2	1547 (30.89)	22.2 ± 9.30	468 (30.25)	837 (54.10)	242 (15.64)	40.346	<.001
3-4	1685 (33.65)	20.1 ± 9.59	405 (24.04)	886 (52.58)	394 (23.38)		
Unknown	1776 (35.46)	20.9 ± 8.90	438 (24.66)	988 (55.63)	350 (19.71)		
Years since diagnosis (years)							
<1	2439 (48.70)	21.6 ± 9.60	696 (28.54)	1282 (52.56)	461 (18.90)	14.135	.007
1-2	1086 (21.69)	20.3 ± 9.11	258 (23.76)	600 (55.25)	228 (20.99)		
≥2	1483 (29.61)	20.6 ± 8.85	357 (24.07)	829 (55.90)	297 (20.03)		
Self-reported health status							
Worse	951 (18.99)	18.7 ± 8.95	190 (19.98)	504 (53.00)	257 (27.02)	83.816	<.001
Moderate	1774 (35.42)	20.4 ± 9.11	430 (24.24)	962 (54.23)	382 (21.53)		
Better	2283 (45.59)	22.5 ± 9.34	691 (30.27)	1245 (54.53)	347 (15.20)		
Hospital stay, (days)							
<7	2726 (54.43)	21.0 ± 9.20	698 (25.61)	1514 (55.54)	514 (18.86)	5.043	.080
≥7	2282 (45.57)	21.0 ± 9.41	613 (26.86)	1197 (52.45)	472 (20.68)		

^1^Others include commercial insurance and no medical insurance.

^2^Medical assistance includes national medical aid, drug donation from enterprises, or participation in clinical trials.

Abbreviations: UEBMI, Urban Employees Basic Medical Insurance; URBMI, Urban and Rural Residents Basic Medical Insurance.

### Financial toxicity

The average score of COST for all patients was 21.0 with a SD 9.30, and 26.18%, 54.13%, and 19.69% of patients were categorized into no FT, mild FT, and moderate or severe FT groups, respectively. Different groups of gender, age, marital status, education, registered residency region, annual household income, household size, medical insurance, medical assistance, cancer type, cancer stage, years since diagnosis, and self-reported health status showed significantly different degrees of FT (*P* < .05; Table 1).

### Shared decision-making

Of 5008 patients, 3943 (78.73%) patients reported better SDM. Patients with higher education level, urban registered residency, higher annual household income, UEBMI, advanced stage, and shorter years since diagnosis trended toward a higher possibility of having better SDM. Patients with stomach and colorectal cancer were more likely to report better SDM (Table 2).

**Table 2.** Shared decision-making among Chinese patients with cancer.

Patient characteristics	Worse SDM	Better SDM	Chi-square	*P*
Total	1065 (21.27)	3943 (78.73)	—	—
Demographics				
Gender				
Male	520 (22.05)	1838 (77.95)	1.647	.199
Female	545 (20.57)	2105 (79.43)		
Age (years)				
18-44	249 (21.16)	928 (78.84)	2.415	.491
45-54	288 (21.65)	1042 (78.35)		
55-64	285 (20.04)	1137 (79.96)		
65-90	243 (22.52)	836 (77.48)		
Marital status				
Married	977 (21.16)	3641 (78.84)	0.426	.514
Single/divorced/widowed	88 (22.56)	302 (77.44)		
Ethnicity				
Han	985 (21.11)	3682 (78.89)	1.052	.305
Minority	80 (23.46)	261 (76.54)		
Education				
College or more	240 (19.00)	1023 (81.00)	10.440	.015
High school	270 (20.58)	1042 (79.42)		
Junior school	316 (21.69)	1141 (78.31)		
Primary school or less	239 (24.49)	737 (75.51)		
Registered residency				
Urban	552 (19.59)	2266 (80.41)	10.832	.001
Rural	513 (23.42)	1677 (76.58)		
Annual household income (1000 yuan)				
<30	323 (23.15)	1072 (76.85)	13.650	.001
30-60	380 (22.81)	1286 (77.19)		
≥60	362 (18.59)	1585 (81.41)		
Household size				
1-2	258 (21.20)	959 (78.80)	5.206	.074
3-4	457 (20.05)	1822 (79.95)		
≥5	350 (23.15)	1162 (76.85)		
Medical insurance^1^				
UEBMI	371 (20.16)	1469 (79.84)	6.012	.049
URBMI	640 (22.38)	2220 (77.62)		
Others	54 (17.53)	254 (82.47)		
Medical assistance^2^				
Yes	114 (21.63)	413 (78.37)	0.047	.828
No	951 (21.22)	3530 (78.78)		
Clinical information				
Cancer type				
Lung	182 (21.41)	668 (78.59)	24.010	.004
Esophageal	51 (30.91)	114 (69.09)		
Stomach	75 (20.78)	286 (79.22)		
Colorectal	107 (20.19)	423 (79.81)		
Liver	41 (21.47)	150 (78.53)		
Breast	140 (21.08)	524 (78.92)		
Cervical	70 (24.65)	214 (75.35)		
Other	309 (19.07)	1311 (80.93)		
Unidentified	66 (28.82)	163 (71.18)		
Benign tumor	24 (21.05)	90 (78.95)		
Cancer stage				
1-2	311 (20.10)	1236 (79.90)	14.631	.001
3-4	324 (19.23)	1361 (80.77)		
Unknown	430 (24.21)	1346 (75.79)		
Years since diagnosis (years)				
<1	472 (19.35)	1967 (80.65)	14.090	.001
1-2	231 (21.27)	855 (78.73)		
≥2	362 (24.41)	1121 (75.59)		
Self-reported health status				
Worse	217 (22.82)	734 (77.18)	2.895	.235
Moderate	385 (21.70)	1389 (78.90)		
Better	463 (20.28)	1820 (79.72)		
Hospital stay (days)				
<7	589 (21.61)	2137 (78.39)	0.415	.519
≥7	476 (20.86)	1806 (79.14)		

^1^Others include commercial insurance and no medical insurance.

^2^Medical assistance includes national medical aid, drug donation from enterprises, or participation in clinical trials.

Abbreviations: UEBMI, Urban Employees Basic Medical Insurance; URBMI, Urban and Rural Residents Basic Medical Insurance; SDM, shared decision-making.

### Association between SDM and FT

Multinomial logistic regression was conducted to identify the association between SDM and FT. Compared with their counterparts, patients with better SDM were 50% less likely to have mild FT (adjusted OR 0.50, 95% CI 0.39-0.56), 37% less likely to have moderate or severe FT (adjusted OR 0.63, 95% CI 0.50-0.80; Table 3). For sensitivity analysis, a multivariate linear regression with COST score as the dependent variable was conducted. All VIFs were below 10. The *R*^2^ of the model was 0.1706, and the regression coefficient of SDM is 1.34 (95% CI 0.85-1.83; Table 4). That is, patients experiencing better SDM had an average 1.34 higher score of COST, which indicated lower FT.

**Table 3.** Regression of SDM on FT status.

Model	FT status		
	No	Mild	Moderate or severe
Model 1	1.00	0.47 (0.39, 0.56)^***^	0.57 (0.46, 0.71)^***^
Model 2	1.00	0.50 (0.42, 0.61)^***^	0.63 (0.50, 0.80)^***^

Multinomial logistic regression analyses were used. Model 1, only included SDM variable; model 2, included SDM variable, and adjusted gender, age, marital status, ethnicity, education, registered residency region, annual household income, household size, medical insurance, medical assistance, cancer type, cancer stage, years since diagnosis, self-reported health status, and hospital stay covariates. 95% CIs in brackets. * *P* < .05, ** *P* < .01, *** *P* < .001.

**Table 4.** Regression of SDM on COST score.

Model	β	*t*	*P*	*F*	*R* ^2^
Model 1	1.92 (1.30, 2.55)	6.01	<.001	36.13	0.0072
Model 2	1.34 (0.85, 1.83)	5.32	<.001	32.29	0.1706

Linear regression analyses were used. Model 1, only included SDM variable; model 2, included SDM variable, and adjusted gender, age, marital status, ethnicity, education, registered residency region, annual household income, household size, medical insurance, medical assistance, cancer type, cancer stage, cancer duration, self-reported health status, and hospital stay covariates. Higher COST score indicates lower FT. 95% CIs in brackets. *P < .05, **P < .01, ***P < .001.

Besides, being male, being single/divorced/widowed, lower education level, rural registered residency, lower annual household income, bigger household size, receiving medical assistance, advanced or unknown cancer stage, longer years since diagnosis, worse self-reported health status, and shorter hospital stay were risk factors of FT; older age (between 65 and 90 years old) and with a diagnose of benign tumor were protective factors of FT (Supplementary Table S1).

### Coping actions

**Figure 2.**
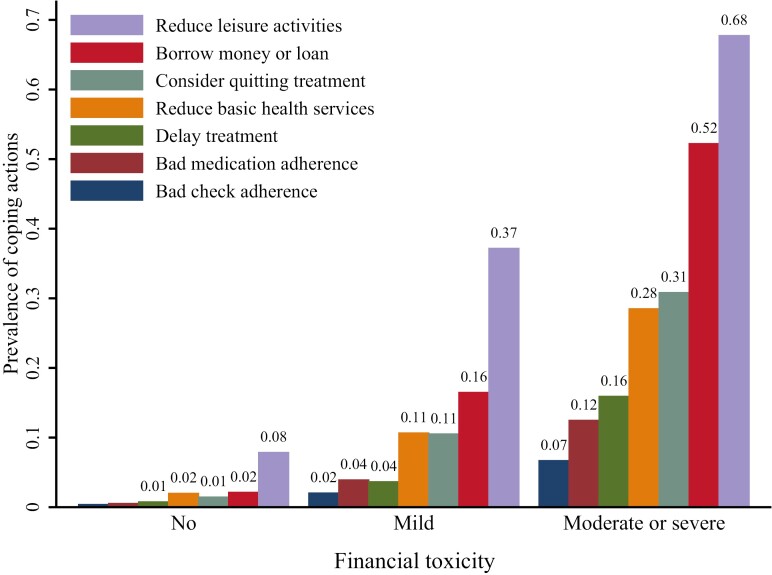
Prevalence of coping actions by different FT groups.

**Figure 3.**
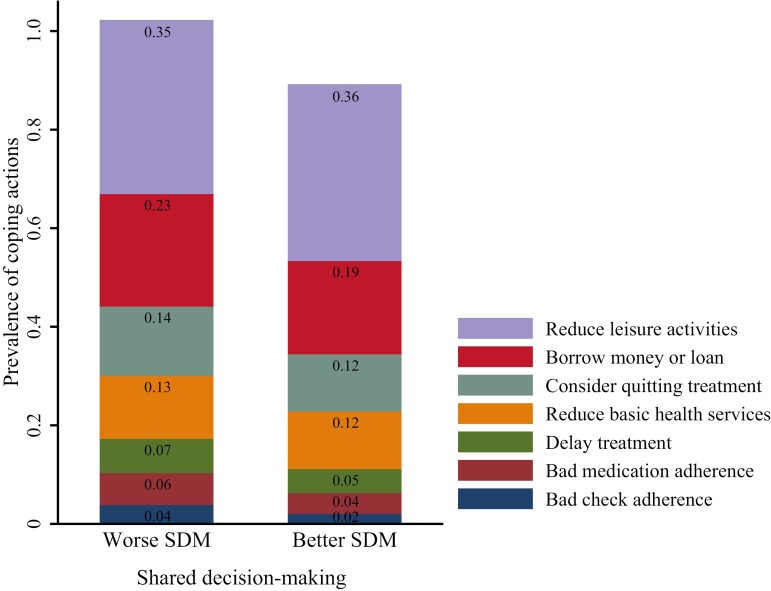
Prevalence of coping actions by different SDM groups.

Of 5008 patients, 12.14% of patients once considered quitting treatment, 5.31% delayed their treatment, 4.71% failed to take medicine as instructed, and 2.52% did not conduct medical checks as instructed. Besides, 35.52% of patients reduced their leisure activity expense, 11.9% reduced their basic health service expense, and 19.77% borrowed money from relatives and friends or acquired a loan from a bank. Compared with patients without FT, patients with higher FT were more likely to take adverse coping actions (all *P* < .001; Figure 2). Compared with those with better SDM, patients with worse SDM were more likely to take some coping actions (*P* < .05) except reducing leisure activity expense and basic health service expense (Figure 3).

## Discussion

This is a national cross-sectional study conducted in 2021, covering over 5000 patients with cancer in 33 public tertiary cancer hospitals across China. Among surveyed patients, over half of the patients had mild FT, and about one-fifth had moderate or severe FT, which indicated a high prevalence of FT among Chinese patients with cancer. From the patient’s perspective, 78.73% of patients reported a good experience with SDM, including clinicians explaining treatment schemes to them and respecting their treatment preference. This study identified positive associations of better SDM and lower FT as well as coping actions and highlighted the importance of implementing SDM in the clinical practice of cancer care. This study also described patient characteristics of those reporting worse SDM and higher FT, which were useful for early screening and intervention among at-risk patients.

### SDM level among Chinese patients with cancer

Patient engagement in decision-making has gradually become popular in clinical practice in recent years and substitutes the long-established physician-led decision-making in China, accompanied with the development of patient-centered value. However, related researches on SDM in China are badly deficient, and the first study on the clinical practice of SDM was in 2015.^28^ In our study, 78.7% of patients reported a good perception of SDM during their hospital stay. A study conducted at 16 hospitals in China in 2019-2020 identified that 73.8% of patients reported a good perceived decision participation among their cancer care, which is relatively consistent with our study although different patient-reported measures were used.^29^ A systematic review summarized various instruments of assessing the process of SDM during surgical consultations and found the results from patient- or clinician-reported measures were higher than those from observer-rated measures, in which patient- or clinician-reported SDM rate of 54%-93% compared with observer-rated SDM of 7%-39%.^30^ A review also revealed that patient-perceived SDM showed association with patient outcomes than observer-rated SDM, and highlighted the importance of understanding the patient’s perspective as critical to the science of measuring SDM.^31^

### Associations of SDM with FT and coping actions

This study confirmed significant associations of SDM with mitigating FT and adverse coping actions in the clinical practice of cancer care. The positive association may derive from multiple impact pathways. For one thing, through involving patients in treatment decision, clinicians tended to adjust medical schemes so as to balance curative effect and medical cost.^24,32^ For another thing, deep communication and considerable caring help in relieving patient’s stress and worries. A study among 247 adolescent and young adult cancer survivors also found cost discussions were related to long-term FT.^33^ Overall, SDM has a crucial impact on the perceived burden and subsequent behavior changes of patients, our study enhances the evidence from patients with cancer in China.

### Patient characteristics associated with SDM

SDM is crucial and attracts much attentions in recent years, while health care providers and patients both face barriers to implement or participate SDM in clinical practice.^34-36^ This study preliminarily explored some patient-level risk factors on the implementation of SDM in clinical settings. Patients having lower socioeconomic status were less engaged in SDM, including those with rural registered residency, lower annual income, and lower education level, which is consistent with previous studies.^37^ Patients with better socioeconomic status tend to have higher health literacy, thus they could better understand clinical information and prefer to participate in decision-making and gain more decision support.^38^ Improving health literacy is a continuous health undertaking, but health care providers should consider to give more patience to these at-risk groups today. Besides, for patients with longer years since diagnosis and at the early cancer stage in our study, their SDM and decision support were overlooked in clinical practice. Some studies found clinicians and health care providers in China and other countries showed positive and supportive attitude toward SDM and cost discussion, while they also expressed their difficulties in acquiring cost information and optimizing communication skills.^39-41^ The need for SDM training program and cost information support was urged, and these should be considered in health system design.

### Patient characteristics associated with FT

Finding characteristics of patients prone to FT is of great value for early screening and taking measures to mitigate FT and accompanying adverse coping actions. Male and middle-aged patients tend to encounter greater financial difficulties because they are usually the main breadwinner of the family, and loss of productivity following cancer leads to a sharp drop in household income.^42^ The expensive medical cost often affects not only patients but also their entire family. Different family structures present contrasting ability to fight economic shock. Our studies found patients with non-married status or large household size perceived greater financial stress. Socioeconomic status is the most direct indicator for perceived FT. Patients with lower education level, lower household income, rural registered residency, and urban and rural residents basic medical insurance were more likely to have higher FT. These were demonstrated in this study and in previous studies.^14,16^

Clinical factors related to health care costs are vital indicators to recognize patients’ FT.^12,43^ In this study, patients with advanced cancer, longer years since diagnosis, and worse self-reported health status may have severe FT, and patients with benign tumors may have a lighter FT. Besides, receiving medical assistance is a risk factor in FT, which is a reverse causation. In clinical practice, government personnel and health care providers evaluate the economic status of patients with cancer, identify high-risk groups to aid. Therefore, patients involved in the medical assistance had a heavier financial burden in this study, which to some extent reflected the rationality and precision of selection of aid objects.

### Strengths and limitations

This study has some strengths. This study is a nationwide multicenter survey focusing on SDM as well as FT in cancer clinical practice, and verifying the positive association of SDM on alleviating FT, which adds to crucial evidence for international fields. Meanwhile, patient characteristics of higher FT and worse SDM were described to facilitate targeted focus and intervention for at-risk groups. Potential coping actions patients may take when facing financial burden indicated targeted points to monitor and administrate for oncologists and health system.

Some limitations also existed in this study. First, this is a cross-sectional survey and causality cannot be inferred. This study evaluated one SDM process during one hospitalization. Although it is acceptable and common in the cross-sectional survey, a longitudinal study with repeated-measures will add more strengths. Second, there is no widely accepted tool for measuring the process of SDM.^30,44^ Our study quantifies SDM using 2 patient-reported items, which takes patient-centered values into consideration and has its strength. At the same time, it is a pity that the types and contents of decision were not examined in our study and their effect was unexplored. The more pinpoint-designed tool and precise measures remain to be conducted in the future. Third, this study has recall bias due to the self-reported data source. The recall bias was somewhat limited because we investigated patients who just near discharge and they had relatively clearer memory about the hospitalization experience. Fourth, detailed treatment schemes information was lacking, thus the effect of treatment schemes on FT was not analyzed. Linking surveyed data with electronic medical records helps to carry out more in-depth exploration in the future. Finally, due to the limitation of time and cost, this study enrolled only public tertiary cancer hospitals, not private and other public hospitals, which weakened the representation of the study population to some extent. This may lead to relatively high SDM and FT levels because these hospitals usually have better service strengths and accommodate patients with severe cancer.

## Conclusions

This study conducted in China described the prevalence of SDM, FT and coping actions among adult patients with cancer, and further explored the impact of SDM on FT, as well as identified patient characteristics prone to FT. These findings contribute to potential intervention points at the level of health system, oncologists, and patients. A more supportive environment in future is needed so as to help us find at-risk groups, promote SDM between oncologists and patients with cancer, alleviate the effect of FT, and improve the experience of cancer care and health outcomes for patients with cancer. Besides, more in-depth studies remain to be conducted in the future and take the extent and quality of SDM as well as long-term FT into consideration.

## Supplementary Material

oyae065_suppl_Supplementary_Table_S1

## Data Availability

The data that support the findings of this study are available from the corresponding author upon reasonable request.
