# Supplementary material for: Association of shared decision-making with financial toxicity and coping actions in adult patients with cancer: a nationwide study in China
Source: Oncologist. 2024 Sep 1;29(8):e1031–40. doi: 10.1093/oncolo/oyae065 (PMC11365698; doi:10.1093/oncolo/oyae065)
Supplement: oyae065_suppl_Supplementary_Table_S1 [file oyae065_suppl_supplementary_table_s1.docx]

Supplementary material

Table S1 **Complete model result on financial toxicity and COST score**

| **Patient characteristics** | **Model A** | | **Model B** |
| --- | --- | --- | --- |
|  | **Mild vs No** | **Moderate or Severe vs No** | ***β*** |
| Worse SDM | 1.00 | 1.00 | 1.00 |
| Better SDM | 0.50(0.42,0.61)^***^ | 0.63(0.50,0.80)^***^ | 1.34(0.85,1.83)^***^ |
| **Demographics** |  |  |  |
| Gender |  |  |  |
| Male | 1.36(1.16,1.59)^***^ | 1.26(1.03,1.55)^*^ | -0.82(-1.38,-0.27)^**^ |
| Female | 1.00 | 1.00 | 1.00 |
| Age,years |  |  |  |
| 18-44 | 1.00 | 1.00 | 1.00 |
| 45-54 | 0.91(0.74,1.11) | 1.18(0.90,1.54) | -0.25(-0.95,0.45) |
| 55-64 | 0.82(0.66,1.02) | 0.90(0.68,1.19) | 0.29(-0.44,1.03) |
| 65-90 | 0.71(0.56,0.90)^**^ | 0.63(0.46,0.87)^**^ | 1.41(0.61,2.21)^***^ |
| Marital status |  |  |  |
| Married | 1.00 | 1.00 | 1.00 |
| Single/divorced/widowed | 1.08(0.82,1.42) | 1.53(1.10,2.15)^*^ | -1.10(-2.02,-0.19)^*^ |
| Ethnicity |  |  |  |
| Han | 1.00 | 1.00 | 1.00 |
| Minority | 1.08(0.80,1.44) | 1.02(0.71,1.47) | -0.01(-0.91,0.88) |
| Education |  |  |  |
| College or more | 1.00 | 1.00 | 1.00 |
| High school | 1.39(1.14,1.70)^**^ | 1.65(1.24,2.18)^***^ | -1.47(-2.18,-0.77)^***^ |
| Junior school | 1.43(1.15,1.78)^**^ | 1.76(1.31,2.36)^***^ | -1.78(-2.54,-1.01)^***^ |
| Primary school or less | 1.50(1.14,1.96)^**^ | 1.62(1.14,2.31)^**^ | -1.77(-2.66,-0.88)^***^ |
| Registered residency region |  |  |  |
| Urban | 1.00 | 1.00 | 1.00 |
| Rural | 1.15(0.96,1.37) | 1.52(1.21,1.90)^***^ | -1.17(-1.75,-0.59)^***^ |
| Annual household income (1000 yuan) |  |  |  |
| <30 | 2.79(2.28,3.42)^***^ | 7.55(5.80,9.81)^***^ | -5.80(-6.46,-5.14)^***^ |
| 30-60 | 2.16(1.82,2.55)^***^ | 4.37(3.45,5.52)^***^ | -4.25(-4.84,-3.66)^***^ |
| ≥60 | 1.00 | 1.00 | 1.00 |
| Household size |  |  |  |
| 1-2 | 1.00 | 1.00 | 1.00 |
| 3-4 | 1.25(1.04,1.50)^*^ | 1.47(1.16,1.88)^**^ | -1.30(-1.94,-0.67^)***^ |
| ≥5 | 1.20(0.99,1.47) | 1.48(1.15,1.92)^**^ | -1.12(-1.79,-0.45)^**^ |
| Medical insurance† |  |  |  |
| UEBMI | 1.00 | 1.00 | 1.00 |
| URBMI | 1.01(0.85,1.21) | 1.06(0.84,1.34) | 0.11(-0.49,0.71) |
| Others | 0.77(0.58,1.02) | 0.84(0.56,1.26) | 0.53(-0.53,1.58) |
| Medical assistance^‡^ |  |  |  |
| Yes | 1.25(0.97,1.61) | 1.60(1.18,2.15)^**^ | -1.75(-2.50,-1.00)^***^ |
| No | 1.00 | 1.00 | 1.00 |
| **Clinical information** |  |  |  |
| Cancer type |  |  |  |
| Lung | 1.00 | 1.00 | 1.00 |
| Esophageal | 0.72(0.47,1.08) | 0.51(0.29,0.89)^*^ | 0.60(-0.70,1.89) |
| Stomach | 0.98(0.72,1.33) | 0.65(0.42,0.98)^*^ | 0.66(-0.40,1.71) |
| Colorectal | 1.02(0.78,1.35) | 0.89(0.62,1.26) | 0.01(-0.92,0.95) |
| Liver | 0.93(0.60,1.42) | 1.17(0.71,1.93) | -0.93(-2.15,0.28) |
| Breast | 0.99(0.75,1.32) | 1.02(0.71,1.46) | -0.30(-1.28,0.67) |
| Cervical | 0.85(0.59,1.22) | 0.57(0.35,0.90)^*^ | 1.16(-0.08,2.40) |
| Other | 0.81(0.65,1.01) | 0.76(0.58,1.00) | 0.63(-0.13,1.38) |
| Unidentified | 0.85(0.57,1.27) | 0.58(0.35,0.98)^*^ | 1.39(0.14,2.65)^*^ |
| Benign tumor | 0.48(0.30,0.75)^**^ | 0.25(0.12,0.51)^***^ | 4.58(2.57,6.59)^***^ |
| Cancer stage |  |  |  |
| 1-2 | 1.00 | 1.00 | 1.00 |
| 3-4 | 1.08(0.91,1.29) | 1.54(1.23,1.94)^***^ | -1.17(-1.79,-0.55)^***^ |
| Unknown | 1.25(1.05,1.49)^*^ | 1.61(1.28,2.04)^***^ | -1.28(-1.88,-0.69)^***^ |
| Years since diagnosis, years |  |  |  |
| <1 | 1.00 | 1.00 | 1.00 |
| 1-2 | 1.19(1.00,1.43) | 1.24(0.98,1.57) | -0.81(-1.42,-0.20)^**^ |
| ≥2 | 1.17(0.99,1.39) | 1.16(0.93,1.45) | -0.69(-1.26,-0.11)^*^ |
| Self-reported health status |  |  |  |
| Worse | 1.50(1.23,1.84)^***^ | 2.69(2.10,3.44)^***^ | -3.30(-3.95,-2.65)^***^ |
| Moderate | 1.30(1.11,1.51)^**^ | 1.86(1.52,2.29)^***^ | -2.01(-2.55,-1.48)^***^ |
| Better | 1.00 | 1.00 | 1.00 |
| Hospital stay, day |  |  |  |
| <7 | 1.22(1.06,1.40)^**^ | 1.12(0.93,1.34) | -0.51(-1.00,-0.03)^*^ |
| ≥7 | 1.00 | 1.00 | 1.00 |

Note: Model A, financial toxicity as dependent variable, multinomial logistic regression was used; Model B, COST score as dependent variable, linear regression was used. Model A and B both included shared decision-making variable, and adjusted gender, age, marital status, ethnicity, education, registered residency region, annual household income, household size, medical insurance, medical assistance, cancer type, cancer stage, years since diagnosis, self-reported health status, and hospital stay covariates.

† Others include commercial insurance and no medical insurance. ‡ Medical assistance includes national medical aid, drug donation from enterprises, or participation in clinical trials. Abbreviations: UEBMI, Urban Employees Basic Medical Insurance; URBMI, Urban and Rural Residents Basic Medical Insurance. 95% confidence intervals in brackets. * *P*<0.05, ** *P*<0.01, *** *P*<0.001.
